# Uptake, acceptability and interpretability of 3‐in‐1 rapid blood self‐testing for HIV, hepatitis B and hepatitis C

**DOI:** 10.1002/jia2.26053

**Published:** 2022-12-23

**Authors:** Nicolas Salvadori, Jullapong Achalapong, Chonlatorn Boontan, Choosakun Piriya, Surachet Arunothong, Sawitree Nangola, Chiraphat Kloypan, Eakkapote Prompunt, Woottichai Khamduang, Phornphimon Moolnoi, Sakorn Pornprasert, Sumet Ongwandee, Jean Yves Mary, Gonzague Jourdain, Nicole Ngo‐Giang‐Huong

**Affiliations:** ^1^ Thailand‐France Research Collaboration, Faculty of Associated Medical Sciences Chiang Mai University Chiang Mai Thailand; ^2^ Department of Statistics, Faculty of Science Chiang Mai University Chiang Mai Thailand; ^3^ Chiangrai Prachanukroh Hospital Chiang Rai Thailand; ^4^ Office of Disease Prevention and Control Region 1 Chiang Mai Department of Disease Control Ministry of Public Health Chiang Mai Thailand; ^5^ Division of Clinical Immunology and Transfusion Sciences Department of Medical Technology School of Allied Health Sciences University of Phayao Phayao Thailand; ^6^ Department of Pathology, School of Medicine University of Phayao Phayao Thailand; ^7^ Division of Clinical Microbiology, Department of Medical Technology School of Allied Health Sciences University of Phayao Phayao Thailand; ^8^ Department of Medical Technology Faculty of Associated Medical Sciences Chiang Mai University Chiang Mai Thailand; ^9^ INSERM U1153, Team ECSTRRA Université Paris Cité, Hôpital Saint‐Louis Paris France; ^10^ MIVEGEC Univ. Montpellier, CNRS, IRD Montpellier France

**Keywords:** hepatitis B, hepatitis C, HIV, point‐of‐care testing, rapid diagnostic test, self‐testing

## Abstract

**Introduction:**

Early diagnosis is key to achieving the goal of eliminating transmission of HIV and hepatitis B and C. We assessed the uptake, acceptability and interpretability of self‐testing using a 3‐in‐1 rapid diagnostic test (RDT) in facility‐based services.

**Methods:**

Stand‐alone testing services were provided free of charge to consenting individuals aged ≥15 years in five facilities in northern Thailand. Clients were invited to choose between self‐testing by fingerprick or venepuncture by a healthcare worker (HCW). In each facility, several clients could simultaneously self‐test in separate private areas using TriQuik™ (Genlantis, San Diego, CA, USA), a single immunochromatographic cassette detecting HIV‐1/2 antibody, hepatitis B surface antigen (HBsAg) and hepatitis C antibody (HCAb). An interactive program on a tablet computer was developed to collect socio‐demographic, behavioural and satisfaction data and provide information to guide the self‐test process, including video instructions, results interpretation and a picture of the cassette for immediate remote review by the HCW. When the HCW interpreted an HIV self‐test as positive, the HCW collected blood by venepuncture for immediate confirmation.

**Results:**

Between October 2020 and April 2022, 4119 clients presented for testing for the first time as part of the project. Of them, 3462 (84.0%) opted for self‐testing. Among self‐testers, 1801 (52.0%) were born female, the median age was 27 years (interquartile range, 22–36), 661 (19.1%) belonged to at least one key population and 2124 (61.4%) had never been tested for HIV; 3329 (99.8% of those who answered) reported being “very satisfied” or “satisfied” with the testing process. The proportions of test results interpreted as positive by self‐testers among those interpreted as positive by HCWs were 95% for HIV‐1/2 antibody, 95% for HBsAg and 78% for HCAb.

**Conclusions:**

These proportions were higher than those observed in a previous study evaluating another 3‐in‐1 RDT for HIV, HBsAg and HCAb, possibly due to the use of video instructions instead of paper‐based instructions, lower prevalence and co‐infection rates, or lower percentages of clients with low education level. Multiplex self‐testing simplified and streamlined the service delivery process and was well accepted. HCW assistance proved to be essential in a limited number of cases.

## 1 INTRODUCTION

Human immunodeficiency virus (HIV), hepatitis B virus (HBV) and hepatitis C virus (HCV) are three viruses of major public health concern, totalling each year an estimated 4.5 million new infections (1.5 million each) and 1.8 million deaths (680,000 due to acquired immunodeficiency syndrome, 820,000 to hepatitis B and 290,000 to hepatitis C) [1, 2]. Although awareness of infection status is key for prevention and treatment interventions, only 84% of people with HIV, 10% with chronic hepatitis B and 21% with chronic hepatitis C know their status [1, 2].

Innovative approaches to testing services are needed to reach infected individuals not aware of their status who would benefit from existing treatments. One is self‐testing, the process in which individuals collect their own specimen, perform a rapid diagnostic test (RDT) and interpret the result. Self‐testing has been found a convenient and discreet approach to testing and has been recommended by the World Health Organization (WHO) since 2016 for HIV [3] and since 2021 for HCV [4]. Another approach is multiplex testing, which consists of simultaneously testing a sample for multiple infections. However, to date, multiplex tests are not widely available. The only multiplex tests in the WHO list of prequalified in vitro diagnostic products are dual HIV/syphilis RDTs [5], in line with WHO recommendations to use such tests for pregnant women in antenatal care [6]. Multiplex self‐testing may contribute to increasing the uptake of regular HIV, HBV and HCV screening by at‐risk individuals and thus increasing rates of access to diagnosis [2, 4, 7], as well as decreasing testing‐related costs and optimizing time spent by healthcare workers (HCWs) and clients [8].

We assessed the uptake, acceptability and interpretability of a new 3‐in‐1 rapid blood self‐testing for HIV, HBV and HCV offered as part of facility‐based testing services in the context of a research project.

## 2 METHODS

### 2.1 Setting and population

Stand‐alone testing services for HIV, HBV and HCV were provided free of charge to consenting individuals aged at least 15 years in five facilities in northern Thailand—three in Chiang Mai, one in Chiang Rai and one in Phayao—as part of the “Napneung” research project (ClinicalTrials.gov: NCT04585165).

### 2.2 Service delivery process

After completing a socio‐demographic and behavioural questionnaire on a tablet computer, clients were invited to choose between self‐testing by fingerprick or venepuncture by an HCW—the standard blood collection process in Thailand—using TriQuik™ (Genlantis, San Diego, CA, USA), an immunochromatographic RDT for the detection of HIV‐1/2 antibody, hepatitis B surface antigen (HBsAg) and hepatitis C antibody that can be used with whole blood [9]. In separate areas ensuring client privacy, several clients could simultaneously self‐test, following instructions provided by an interactive information technology program running on a tablet computer. The self‐sampling process was described step by step with several short videos and the client could pause the program at any time or request the help of the HCW. Fifteen minutes later, clients followed instructions on the tablet computer to interpret the results, took a picture of the test cassette immediately made available on the HCW's tablet computer for remote review and reported their satisfaction level through a 4‐point Likert visual analogue scale. During the waiting time to obtain the test results, the program delivered information about various aspects of the infections of interest [10]. The program interface was available in the three main local languages (Thai, Shan and Burmese) and English. When the HCW interpreted an HIV self‐test as positive, the HCW collected blood by venepuncture for immediate confirmation with two other antibody tests: SD Bioline^TM^ HIV‐1/2 3.0 (Standard Diagnostics, Yongin, South Korea) [11] and ONE STEP Anti‐HIV (1&2) Tri‐line (InTec, Xiamen, China) [5]. All discrepancies in the interpretation of the self‐test results between the client and the HCW were independently reviewed by a second HCW retrospectively. Clients with any positive test received post‐test counselling and immediate personalized referral for evaluation and treatment.

### 2.3 Statistical analyses

Clients’ self‐reported characteristics were summarized using descriptive statistics. Clients were considered as belonging to a “key population” [12] if they reported being born male and having ever had sex with a man, having received benefits in exchange of sex within the past 6 months or having injected drugs within the past 6 months.

The proportion of test results interpreted as positive by self‐testers among those interpreted as positive by HCWs and the proportion of test results interpreted as negative by self‐testers among those interpreted as negative by HCWs were calculated. The 95% confidence intervals (CIs) for these proportions were calculated using the Clopper–Pearson method [13].

The proportion of test results interpreted as positive by HCWs among those interpreted as positive by self‐testers and the proportion of test results interpreted as negative by HCWs among those interpreted as negative by self‐testers were calculated for prevalence rates ranging from 0.5% to 10% [14, 15, 16]. The 95% CIs for these proportions were calculated using the standard logit method [17].

The association between self‐testers’ characteristics and misinterpretation of at least one result was assessed using Fisher's exact test or Wilcoxon–Mann–Whitney test as appropriate. Statistical analyses were performed using Stata 16.1 (StataCorp, College Station, TX, USA).

Before the introduction of TriQuik^TM^, clients presenting for testing as part of the project underwent venepuncture by an HCW and were tested for HIV‐1/2 antibodies using Alere^TM^ HIV Combo (Alere Medical Co., Ltd., Chiba, Japan), HBsAg using Alere Determine^TM^ HBsAg (Alere Medical Co., Ltd.) and hepatitis C antibodies using SD Bioline^TM^ HCV (Standard Diagnostics). We evaluated the sensitivity and specificity of TriQuik^TM^ against these gold standard tests in plasma samples of a subset of clients randomly selected among those with at least one positive test.

### 2.4 Ethical considerations

The protocol was approved by the national ethics committee of the Institute for the Development of Human Research Protections at the Thailand Ministry of Public Health and by ethics committees at the involved institutions. Informed consent was obtained from each participant.

## 3 RESULTS

### 3.1 Pre‐study TriQuik^TM^ performance assessment

Between 19 October 2015 and 8 November 2019, 469 clients had a positive test for HIV‐1/2 antibodies, HBsAg and/or hepatitis C antibodies. Of them, 52 had their plasma sample retrospectively tested with TriQuik^TM^ in March 2020. TriQuik^TM^ sensitivity and specificity were, respectively, 97% (30/31; 95% CI, 83%–>99%) and 90% (19/21; 70–99%) for HIV‐1/2 antibodies; 100% (17/17; 81–100%) and 94% (33/35; 81–99%) for HBsAg; and 88% (14/16; 62–98%) and 94% (34/36; 81–99%) for hepatitis C antibodies. Following these results, the three individual tests were replaced with TriQuik^TM^.

### 3.2 Study population and uptake of multiplex self‐testing

Between 19 October 2020 and 7 April 2022, 4119 clients presented for testing for the first time as part of the project. Of them, 3462 (84.0%) opted for self‐testing. Based on HCWs’ feedback, the main reason for not opting for self‐testing was the fear of pressing the lancet against the fingertip. A description of clients’ self‐reported characteristics is provided in Table 1. Twenty HCWs participated in the study and the median number of clients per HCW during the study period was 92 (IQR, 47–186).

**Table 1:** Clients’ self‐reported characteristics

Clients’ self‐reported characteristics	Self‐testers *N* = 3462 *n* (%) or median (IQR)	Non‐self‐testers *N* = 657 *n* (%) or median (IQR)	Overall *N* = 4119 *n* (%) or median (IQR)
Female sex at birth	1801 (52.0%)	341 (52.0%)	2142 (52.0%)
Age (years)	27 (22–36)	27 (22–35)	27 (22–35)
Never previously tested for HIV	2124 (61.4%)	384 (58.4%)	2508 (60.9%)
Belonging to at least one key population	661 (19.1%)	149 (22.7%)	810 (19.7%)
Cisgender man who has ever had sex with a man	543 (15.7%)	124 (18.9%)	667 (16.2%)
Transgender woman who has ever had sex with a man	25 (0.7%)	7 (1.1%)	32 (0.8%)
Received benefits in exchange of sex within the past 6 months	102 (2.9%)	23 (3.5%)	125 (3.0%)
Injected drugs within the past 6 months	18 (0.5%)	1 (0.2%)	19 (0.5%)

Abbreviations: HIV, human immunodeficiency virus; IQR, interquartile range.

### 3.3 Acceptability of multiplex self‐testing

Of 3337 self‐testers who completed the satisfaction survey, 3061 (91.7%) reported being “very satisfied” with the testing process, 268 (8.0%) “satisfied,” five (0.1%) “dissatisfied” and three (0.1%) “very dissatisfied.”

### 3.4 Interpretability of multiplex self‐testing

Data on positivity rates and accuracy of test results interpretation by self‐testers versus HCWs are provided in Table 2. For all discrepancies, the second HCW's independent retrospective interpretation was consistent with the first HCW's interpretation.

**Table 2:** Accuracy of test results interpretation by self‐testers versus by healthcare workers

Diagnostic test	HIV‐1/2 antibody test	Hepatitis B surface antigen test	Hepatitis C antibody test
Positivity rate^a^	59^b^/3462: 1.7%	98/3462: 2.8%	9/3462: 0.3%
Proportion of test results interpreted as positive by self‐testers among those interpreted as positive by HCWs (95% CI)	56/59: 95% (86–100%)	93/98: 95% (88–98%)	7/9: 78% (40–97%)
Proportion of test results interpreted as negative by self‐testers among those interpreted as negative by HCWs (95% CI)	3325/3403: 97.7% (97.1–98.2%)	3329/3364: 99.0% (98.6–99.3%)	3418/3453: 99.0% (98.6–99.3%)
Proportion of test results interpreted as positive by HCWs among those interpreted as positive by self‐testers (95% CI) for a range of prevalence levels			
0.5% prevalence	17.2% (14.2–20.7%)	31.4% (24.7–39.0%)	27.8% (19.3–38.4%)
1% prevalence	29.5% (25.0–34.4%)	48.0% (39.8–56.2%)	43.7% (32.4–55.6%)
2% prevalence	45.8% (40.2–51.5%)	65.1% (57.2–72.2%)	61.0% (49.2–71.7%)
5% prevalence	68.5% (63.5–73.2%)	82.8% (77.5–87.0%)	80.2% (71.4–86.7%)
10% prevalence	82.1% (78.6–85.2%)	91.0% (87.9–93.4%)	89.5% (84.1–93.2%)
Proportion of test results interpreted as negative by HCWs among those interpreted as negative by self‐testers (95% CI) for a range of prevalence levels			
0.5% prevalence	100.0% (99.9–100.0%)	100.0% (99.9–100.0%)	99.9% (99.6–100.0%)
1% prevalence	99.9% (99.8–100.0%)	99.9% (99.9–100.0%)	99.8% (99.2–99.9%)
2% prevalence	99.9% (99.7–100.0%)	99.9% (99.8–100.0%)	99.5% (98.5–99.9%)
5% prevalence	99.7% (99.2–99.9%)	99.7% (99.4–99.9%)	98.8% (96.1–99.7%)
10% prevalence	99.4% (98.3–99.8%)	99.4% (98.7–99.8%)	97.6% (92.2–99.3%)

Abbreviations: CI, confidence interval; HCW, healthcare worker; HIV, human immunodeficiency virus.

^a^ Forty‐four, 29 and seven clients were previously unaware of their HIV, hepatitis B and hepatitis C infection status, respectively. There were two co‐infections: one self‐tester had positive tests for HIV and hepatitis B and one for HIV and hepatitis C (both were unaware of both infections).

^b^ Fifty‐six had positive test results with the two confirmatory antibody tests, one had discrepant results between these two tests (weakly positive with the ONE STEP Anti‐HIV (1&2) Tri‐line and negative with SD Bioline HIV‐1/2 3.0) but had detectable antiretroviral drug concentrations in a subsequent investigation, and two were not tested with the confirmatory antibody tests (both were previously aware of their HIV status).

Overall, 96 (2.8%) self‐testers misinterpreted at least one result: 25 misinterpreted all three results, 12 misinterpreted two results and 59 misinterpreted one result (Table 3). The distribution of sex, age and previous experience of HIV testing did not significantly differ from that in self‐testers who correctly interpreted all three results (*p* = 0.84, 0.20 and 0.46, respectively).

**Table 3:** Test results misinterpreted by self‐testers

**Self‐tester's interpretation**			**Healthcare worker's interpretation**			
**HIV‐1/2 antibody**	**Hepatitis B surface antigen**	**Hepatitis C antibody**	**HIV‐1/2 antibody**	**Hepatitis B surface antigen**	**Hepatitis C antibody**	**Number of self‐testers**
**Positive**	Negative	Negative	Negative	Negative	Negative	47
**Positive**	**Positive**	**Positive**	Negative	Negative	Negative	14
**Missing**	**Missing**	**Missing**	Negative	Negative	Negative	7
**Invalid**	**Invalid**	**Invalid**	Negative	Negative	Negative	4
**Positive**	**Positive**	Negative	Negative	Negative	Negative	3
Positive	**Positive**	**Positive**	Positive	Negative	Negative	3
Negative	**Negative**	Negative	Negative	Positive	Negative	3
Negative	**Positive**	**Positive**	Negative	Negative	Negative	2
Negative	Negative	**Negative**	Negative	Negative	Positive	2
Positive	Negative	**Positive**	Positive	Negative	Negative	2
**Negative**	**Positive**	Negative	Positive	Negative	Negative	1
**Positive**	**Negative**	Negative	Negative	Positive	Negative	1
**Positive**	Negative	**Positive**	Negative	Negative	Negative	1
**Positive**	Positive	**Positive**	Negative	Positive	Negative	1
**Negative**	Negative	Negative	Positive	Negative	Negative	1
**Positive**	Positive	Negative	Negative	Positive	Negative	1
Negative	**Positive**	Negative	Negative	Negative	Negative	1
Positive	**Positive**	Negative	Positive	Negative	Negative	1
Positive	Positive	**Positive**	Positive	Positive	Negative	1

Note: Test results misinterpreted by self‐testers are in bold.

Abbreviation: HIV, human immunodeficiency virus.

## 4 DISCUSSION

In this real‐world study among more than 4000 clients presenting for testing in northern Thailand, the proportion of test results interpreted as negative by self‐testers among those interpreted as negative by HCWs was high, with 95% CI lower bounds above 97% for all three tests. The proportion of test results interpreted as positive by self‐testers among those interpreted as positive by HCWs was above 90% for all tests except for hepatitis C antibody (only nine positive tests), but the low prevalence rates observed in our study did not allow for precise estimates. In a systematic review and meta‐analysis, HIV RDT results interpreted by self‐testers were also found highly concordant with those interpreted by HCWs [18], and our estimates are consistent with those found in these studies. In a study in the Democratic Republic of the Congo where another 3‐in‐1 RDT for HIV, HBV and HCV was used, the proportion of test results interpreted as negative by self‐testers among those interpreted as negative by HCWs was 92% (95% CI, 87–95%) for HIV, 96% (92–98%) for HBV and 89% (84–93%) for HCV, and the proportion of test results interpreted as positive by self‐testers among those interpreted as positive by HCWs was 71% (53–85%) for HIV, 73% (57–85%) for HBV and 86% (42–99%) for HCV [19]. The higher rates observed in our study may be due to the use of video instructions instead of paper‐based instructions, lower prevalence and co‐infection rates, or lower percentages of clients with low education level or residing in rural areas [19]. In our study, the proportion of test results interpreted as positive by HCWs among those interpreted as positive by self‐testers was low for all three tests, emphasizing the need for HCW's review of test results interpreted as positive by self‐testers, in particular in settings with low prevalence rates. With the wide availability of image recognition techniques, a more promising option would be to develop an algorithm able to automatically interpret the results based on the picture of the test cassette taken by the client.

Our multiplex rapid blood self‐testing process for HIV, HBV and HCV was more frequently chosen than conventional blood sampling by an HCW and was highly accepted. This approach has the potential to decrease time spent by HCWs and clients and its associated costs: the multiplex feature provides the opportunity to screen an individual for several infections—including those that may have never been screened if single tests were used—using only a single drop of blood, and the self‐testing feature provides the opportunity to screen several individuals simultaneously under the remote supervision of a single HCW. Another advantage of the self‐testing feature is that it can be performed without close contact with the HCW, thereby reducing the risk of transmission of SARS‐CoV‐2 or other air‐borne pathogens. The self‐ and multi‐testing process that we evaluated in healthcare facilities in Thailand may be successfully implemented outside healthcare settings, including at home, and in other countries with various epidemiologic situations and socio‐economic context. However, this process may need to be adapted, optimized and evaluated in these different settings. Adding *Treponema pallidum* as part of multiplex testing would be important, in particular in view of achieving the goal of triple elimination of mother‐to‐child transmission of syphilis, HIV and hepatitis B [20, 21].

The main strengths of this study are the large sample size and the fact that it was conducted in a real‐world setting, with no eligibility criteria besides informed consent and age for ethical reasons. The main limitation is that whole blood samples tested with TriQuik^TM^ could not be systematically tested with individual regulatory‐approved rapid tests for each of the three diseases, as a new blood collection would have been inconvenient. As a result, rates of false‐positive results for HBsAg and hepatitis C antibody tests and of false‐negative results for all three tests could not be assessed. However, in our pre‐study assessment, the performance of TriQuik^TM^ using well‐characterized plasma samples was satisfactory and positivity rates were found consistent with those observed in our facilities before the introduction of TriQuik^TM^. Another limitation is that the association between education level and interpretability of multiplex self‐testing could not be assessed because data on education level were not collected. Several studies found that misinterpretation of HIV test results occurred more frequently in self‐testers with lower education level [22, 23]. Finally, co‐infection rates and HCV prevalence rates were very low in our study, thus it is unclear whether the interpretability results of TriQuik™ as self‐testing would be similar in settings with higher prevalence and co‐infection rates [1, 24].

## 5 CONCLUSIONS

Multiplex self‐testing simplified and streamlined the service delivery process and was well accepted. HCW assistance proved to be essential in a limited number of cases.

## COMPETING INTERESTS

The authors declare no competing interests.

## AUTHORS’ CONTRIBUTIONS

NS, GJ and NNGH conceived the study. NS analysed the data and drafted the manuscript. JA, CB, CP, SA, SN, CK, EP, WK, PM, SP, SO, JYM, GJ and NNGH contributed to the interpretation of the results and revised the manuscript. All authors read and approved the final manuscript.

## FUNDING

The Napneung project is supported by a grant from Expertise France through *L'Initiative* (18SANIN210).

## Data Availability

The data that support the findings of this study are available from the corresponding author upon reasonable request.
